# Association of interleukin 16 gene polymorphisms and plasma IL16 level with osteosarcoma risk

**DOI:** 10.1038/srep34607

**Published:** 2016-10-05

**Authors:** Yu-Jin Tang, Jun-Li Wang, Ke-Gong Xie, Chang-Gong Lan

**Affiliations:** 1 Department of Orthopedic Surgery, the Affiliated Hospital of Youjiang Medical University for Nationalities, Baise 533000, Guangxi, China

## Abstract

Interleukin (IL) 16 plays a key role in inflammatory diseases as well as in tumorigenesis of osteosarcoma (OS). The aim of this study was to investigate the association of *IL16* polymorphisms and plasma IL16 level with OS risk in a Chinese population. We genotyped *IL16* rs4778889, rs11556218, and rs4072111 in 358 patients with OS and 402 controls using a polymerase chain reaction-restriction fragment length polymorphism assay. Plasma IL16 level was measured by enzyme-linked immunosorbent assay. Rs11556218 was associated with an increased risk of OS in heterozygote comparison (adjusted OR = 1.65, 95% CI, 1.23–2.21, *P* < 0.001), dominant model (adjusted OR = 1.66, 95% CI, 1.24–2.21, *P* < 0.001), and allele comparison (adjusted OR = 1.44, 95% CI, 1.14–1.81, *P* = 0.002). Moreover, rs11556218 TG/GG genotypes were associated with higher levels of IL16 as compared to TT genotype (*P* = 0.03). However, no significant association of rs4778889 and rs4072111 and OS was found. These findings suggest that rs11556218 TG/GG genotypes may be associated with increased susceptibility to OS, probably by increasing the production of IL16 level.

Osteosarcoma (OS), derived from primitive transformed cells of mesenchymal origin, is an aggressive malignant neoplasm in bone and often occurs in children and young adults^1^,^2^,^3^,^4^. Through improvements of treatment strategies, the 5-year survival rate of localized OS patients has increased to 60–80%, while the survival rate is only 25% in patients with metastases at the time of diagnosis^3^,^5^,^6^,^7^. It is of great importance, therefore, to uncover the molecular mechanism involved in the tumorigenesis of OS. It has been identified that radiation and chemicals exposure are risk factors for the development of OS^8^,^9^. However, only a few individuals exposed to the similar risk factors develop OS, suggesting that OS is a complex, multistep, and multifactorial disease^10^,^11^. Previously, several candidate genes have been reported to influence individuals’ susceptibility to OS, such as glutamate receptor metabotropic 4 gene, interleukin (IL) 1β gene, *IL6*, and *IL12* 12,13,14,15.

IL16 is a pleiotropic cytokine and functions as a modulator not only in inflammatory processes but also in tumorigenesis. By binding to the CD4 molecule, IL16 can activate monocytes and stimulate the secretion of inflammatory cytokines, including tumor necrosis factor-α (TNF-α), IL1β, IL6, and IL15. These cytokines were related to the development of OS^16^,^17^,^18^,^19^,^20^,^21^,^22^,^23^,^24^.

*IL16*, located on chromosome 15q26.3 in the human genome, encodes IL16 cytokine^25^. Recently, association studies have been carried out to investigate the relationship of single nucleotide polymorphisms (SNPs) in *IL16* with risk of a series of cancer types, including colorectal cancer^26^,^27^, gastric cancer^26^,^28^,^29^,^30^, nasopharyngeal carcinoma^31^,^32^, hepatocellular carcinoma^33^, prostate cancer^34^, renal cell carcinoma^35^,^36^, and glioma^37^. No report, nevertheless, was performed to examine the association between three common SNPs (i.e., rs4778889, rs11556218, and rs4072111) in *IL16* and OS risk. In this study, we evaluated the association of the three SNPs in *IL16* with susceptibility to OS in a Chinese population. Moreover, the effect of *IL16* polymorphisms on plasma level of IL16 was also assessed.

## Materials and Methods

### Ethics Statement

The study protocol was approved by the Review Boards of Affiliated Hospital of Youjiang Medical College for Nationalities. The study was carried out in accordance with the relevant guidelines. Informed consent was signed by each adult participant or guardians on the behalf of the children participants.

### Study population

This study population included 358 OS patients and 402 healthy controls from the Affiliated Hospital of Youjiang Medical College for Nationalities between January 2008 and June 2015. Diagnosis of OS was confirmed by histological examination. Clinical information was extracted from medical records, including age of diagnosis, gender, family history of cancer, tumor location, and metastasis. We excluded those patients with a history of familial cancer from this study. The control subjects were recruited from individuals who underwent routine health examination at the same hospital during the same period. The selection criteria for control subjects were as follows: healthy volunteers without OS, hypertension, and diabetes mellitus; no history of any cancer, cardiovascular diseases, and other inflammatory diseases; no family history of any cancer. The controls were frequency matched to cases in terms of age, gender, and residence area. All subjects were unrelated Han Chinese.

### SNPs selection

We selected three SNPs in *IL16*, which have been identified to be functional previously^38^,^39^,^40^: rs4778889 (c. −475 T > C); rs11556218 (c. 3441 T > G; p. Asn1147Lys); and rs4072111 (c. 1300 C > T; p. Pro434Ser).

### Genotyping

Genomic DNA was extracted from peripheral blood using a commercial kit according to the manufacturer’s manuals (Tiangen Inc., Beijing, China). The polymerase chain reaction–restriction fragment length polymorphism (PCR-RFLP) assay was carried out to genotype *IL16* rs4778889, rs11556218, and rs4072111. PCR primer sequences, annealing temperature, restriction enzymes used, and length of PCR products were prepared as described previously^26^. The genotyping results were confirmed by Sanger sequencing.

### Plasma IL16 Level

Blood samples were obtained from patients with OS and healthy controls. After centrifugation at 1000 g for 10 min, the plasma was stored at −80 °C until analysis. Plasma IL16 concentration was measured by enzyme-linked immunosorbent assay (ELISA) (Raybiotech, Norcross, GA, USA) according to the manufacturer’s instructions. The minimum detectable dose of the human IL16 ELISA kit was 5 pg/mL. No cross-reactivity with other cytokines was tested. All the samples were analyzed in duplicate with intra-assay coefficients of variation less than 10%.

### Statistical analysis

All statistical analyses were done using SPSS statistical software package version 13.0 (SPSS Inc., Chicago, IL, USA). Differences of age between the study groups were assessed using the Student’s *t*-test, whereas differences of gender were evaluated using Pearson χ^2^ test. Hardy-Weinberg equilibrium (HWE) of the three polymorphisms in *IL16* was tested using a goodness-of-fit χ^2^ test. Genotype and allele frequencies between cases and controls were compared using the chi-square test. Odds ratios (ORs) and 95% confidence intervals (CIs) were computed using unconditional logistic regression based on age and gender adjustment. Differences of IL16 plasma level among cases and controls were compared using Mann-Whitney U test. Statistical significance was set at *P* < 0.05.

## Results

### Characteristics of the study subjects

The characteristics of patients with OS and controls are summarized in Table 1. The median age in cases was 18.0 years, ranging from 6.0 to 58.0 years. The median age in controls was 20.0 years, ranging from 12.0 to 59.0 years. No significant difference was found between cases and controls with regard to age (*P* = 0.23) and gender (*P* = 0.91). Most of the tumors (72.1%) located on long tubular bones. Only 29.3% patients had tumor metastasis.

### Association of IL16 polymorphisms with OS risk

The distributions of *IL16* rs4778889, rs11556218, and rs4072111 in OS patients and healthy controls are shown in Tables 2 and 3. All the three SNPs genotyped were in HWE among control subjects (*P* > 0.05). The frequencies of rs11556218 TG and TG/GG genotypes were significantly increased in OS patients compared to controls (TG vs. TT: adjusted OR = 1.65, 95% CI, 1.23–2.21, *P* < 0.001; TG/GG vs. TT: adjusted OR = 1.66, 95% CI, 1.24–2.21, *P* < 0.001). Similarly, the frequency of rs11556218 G allele was significantly higher in OS cases compared to controls (G vs. T: adjusted OR = 1.44, 95% CI, 1.14–1.81, *P* = 0.002). However, no significant association between rs4778889 and rs4072111 and OS risk was observed (*P* > 0.05). When stratification analysis was done according to tumor location and metastasis, no significant difference of *IL16* genotype and allele frequencies was detected among any subgroup of OS patients (data not shown). No evidence of linkage disequilibrium was observed for the three SNPs (for rs4778889 and rs11556218, D′ = 0.15; for rs11556218 and rs4072111, D′ = 0.26; and for rs4778889 and rs4072111, D′ = 0.21).

### Plasma IL16 level and polymorphisms

**Figure 1:**
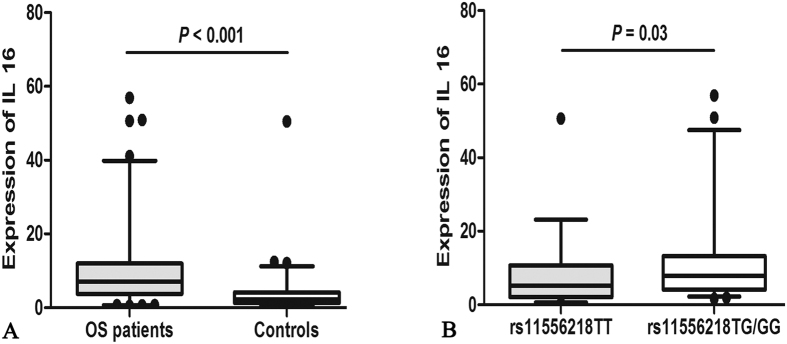
ELISA detection of IL16 expression. (**A**) plasma level of IL16 in osteosarcoma patients (n = 82) and controls (n = 68). (**B**) plasma level of IL16 in patients carrying rs11556218 TG/GG genotypes (n = 46) and patients carrying TT genotype (n = 36). The lines inside the boxes denote the medians. The boxes denote the interval between the 25th and 75th percentiles. The whiskers denote the interval between the 5th and 95th percentiles.

As shown in Fig. 1A, the median plasma IL16 level was 7.02 ng/mL (range 0.48–56.87 ng/mL) in OS patients (n = 82) and 2.21 ng/mL (range 0.09–50.48 ng/mL) in healthy controls (n = 68). The concentration of IL16 in cases was significantly higher than that in controls (*P* < 0.001). However, there was no significant difference of IL16 level among metastatic patients and non-metastatic patients (*P* = 0.65). We further compared the correlation between plasma IL16 level and *IL16* polymorphisms. We found that patients carrying rs11556218 TG/GG genotypes had higher levels of IL16 than those carrying TT genotype (*P* = 0.03, Fig. 1B). Nevertheless, we failed to find any relationship between plasma IL16 level and *IL16* rs4778889 and rs4072111 (*P* > 0.05).

## Discussion

In this hospital-based case-control study, we investigated the association between three common SNPs in *IL16* and risk of OS in a Chinese population. The results revealed that rs11556218 was associated with an increased risk of OS in heterozygote comparison, dominant model, and allele comparison. Moreover, rs11556218 TG/GG genotypes corresponded to higher levels of IL16. These findings indicate that rs11556218 may be responsible for the susceptibility to OS.

The rs11556218, located on exon 6 of *IL16*, can result in an asparagine to lysine substitution. In 2009, Gao *et al*. firstly reported that rs11556218 TG genotype was a risk factor for the development of colorectal cancer and gastric cancer^26^. The positive association was also observed in nasopharyngeal carcinoma^31^,^32^, prostate cancer^34^, and glioma^37^. Meta-analysis conducted by two independent groups confirmed these findings of *IL16* rs11556218 increasing cancer risk^41^,^42^. In agreement with these results, we found that the rs11556218 TG/GG genotypes had a 1.66-fold increased risk of developing OS. We further evaluated whether the genetic polymorphism can influence plasma levels of IL16. Compared with rs11556218 TT genotype, rs11556218 TG/GG genotypes were associated with higher levels of IL16. Taken together, these findings indicate that rs11556218 TG/GG genotypes were risk factors for tumorigenesis of OS, probably by increasing the expression of IL16.

The rs4778889, located at position −295 bp in the promoter region of *IL16*, is related to gene expression and transcriptional activity^38^,^39^,^40^. Previously, the SNP has been studied extensively. Conflicting results, however, were obtained, even in the same ethnic group and cancer type. Some authors reported that *IL16* rs4778889 CC genotype was associated with an elevated risk of non-cardia gastric cancer^28^ and renal cell carcinoma^36^. On the contrary, some authors reported that *IL16* rs4778889 CC genotype was associated with a decreased risk of renal cell carcinoma^35^ and colorectal cancer^27^. Some authors reported that *IL16* rs4778889 was not associated with the risk of colorectal cancer^26^, gastric cancer^26^, nasopharyngeal carcinoma^31^,^32^, and glioma^37^. Our results were consistent with the negative effect. We failed to find any association between *IL16* rs4778889 polymorphism and OS risk, which was verified by three independent meta-analyses^41^,^42^,^43^.

With regard to rs4072111 and cancer risk, contradictory results were also observed^26^,^27^. Some possibilities should be taken into consideration to explain the discrepancy. SNPs have different roles in different cancer types, especially in diverse ethnicities. As for the conflicting results in the same ethnic group and same cancer type, limited sample size and selection bias may be potential reasons. Therefore, population-based association studies with larger sample sizes are necessary to obtain precise results.

IL16, produced by CD8+T cells, can stimulate the secretion of tumor-related cytokines, such as TNF-α, IL1β, IL6, and IL15^16^. Serum level of TNF-α was significantly increased in OS patients, with a function of promoting OS progression^22^,^23^. Anti-TNF-α therapy can inhibit tumor metastasis^24^. IL6 can promote cell motility, proliferation, metastasis, and angiogenesis by inducing the expression of some important genes, such as signal transducer and activator of transcription 3 gene, intercellular adhesion molecule-1 gene, and vascular endothelial growth factor (*VEGF*) gene^44^,^45^,^46^. Knockdown *IL6* can reduce VEGF expression and abolish conditional medium-mediated angiogenesis in human OS cells^46^. Given the key roles of IL16 in OS development, the positive results in this study were biologically reasonable.

Some limitations in this study should be discussed. Both cases and controls were enrolled from a single hospital, which we cannot rule out the possibility of selection bias. However, no deviation from HWE was tested in each SNP, suggesting the possibility is minimal. As is known, OS is composed of complex interactions between genetic and environmental factors. In this study, gene-environment interaction cannot be assessed due to lack of available data. Further gene-environment interaction analysis may provide strong evidence of *IL16* polymorphisms in the etiology of OS. Only OS patients were investigated in this study. Therefore, the results cannot be expanded directly to other pediatric cancers until confirmation was made in different cancer types.

In conclusion, we reported for the first time that *IL16* rs11556218 TG genotype had an elevated risk of OS in the Chinese population. Further well-designed investigations involving in various populations are warranted to verify these results. In the future, further functional analysis of rs11556218 will help us to clarify the potential biological mechanism of OS.

## Additional Information

**How to cite this article**: Tang, Y.-J. *et al*. Association of interleukin 16 gene polymorphisms and plasma IL16 level with osteosarcoma risk. *Sci. Rep*. **6**, 34607; doi: 10.1038/srep34607 (2016).

## Figures and Tables

**Table 1:** General characteristics of the subjects.

Characteristics	Osteosarcoma patients (n = 358)	Controls (n = 402)
Age of diagnosis (median, years)	18.0 (6.0–58.0)	20.0 (12.0–59.0)
Gender, n (%)		
Male	216 (60.3)	241 (60.0)
Female	142 (39.7)	161 (40.0)
Tumor location, n (%)		
Long tubular bones	258 (72.1)	
Axial skeleton	100 (27.9)	
Metastasis, n (%)		
Yes	105 (29.3)	
No	253 (70.7)	

SD, standard deviation.

**Table 2:** Genotype distributions of three SNPs in *IL16* between osteosarcoma patients and controls.

Polymorphisms	Osteosarcoma patients, n = 358 (%)	Controls, n = 402 (%)	Crude OR	Adjusted OR (95% CI)†	Adjusted *P* value†
rs4778889					
TT	215 (60.1)	240 (59.7)	1.00 (Ref)	1.00 (Ref)	
TC	127 (35.5)	140 (34.8)	1.01 (0.75–1.37)	1.01 (0.75–1.37)	0.94
CC	16 (4.5)	22 (5.5)	0.81 (0.42–1.59)	0.81 (0.41–1.60)	0.55
Dominant			0.99 (0.74–1.32)	0.99 (0.74–1.32)	0.92
Recessive			0.81 (0.42–1.56)	0.81 (0.42–1.57)	0.53
rs11556218					
TT	165 (46.1)	235 (58.5)	1.00 (Ref)	1.00 (Ref)	
TG	174 (48.6)	151 (37.6)	**1.64 (1.22–2.20)**	**1.65 (1.23–2.21)**	<0.001
GG	19 (5.3)	16 (4.0)	1.69 (0.84–3.39)	1.69 (0.84–3.40)	0.14
Dominant			**1.65 (1.23–2.19)**	**1.66 (1.24–2.21)**	<0.001
Recessive			1.35 (0.68–2.67)	1.39 (0.70–2.77)	0.34
rs4072111					
CC	218 (60.9)	229 (57.0)	1.00 (Ref)	1.00 (Ref)	
CT	124 (34.6)	158 (39.3)	0.82 (0.61–1.11)	0.82 (0.61–1.11)	0.19
TT	16 (4.5)	15 (3.7)	1.12 (0.54–2.32)	1.12 (0.54–2.33)	0.75
Dominant			0.85 (0.64–1.14)	0.85 (0.63–1.13)	0.26
Recessive			1.21 (0.59–2.48)	1.22 (0.60–2.52)	0.58

SNPs, single nucleotide polymorphisms; OR, odds ratio; CI, confidence interval.

^†^Adjusted by age and gender.

**Table 3:** Allele distributions of three SNPs in *IL16* between osteosarcoma patients and controls.

Polymorphisms	Osteosarcoma patients (%)	Controls (%)	Crude OR	Adjusted OR (95% CI)†	Adjusted *P* value†
rs4778889					
T	557 (77.8)	620 (77.1)	1.00 (Ref)	1.00 (Ref)	
C	159 (22.2)	184 (22.9)	0.96 (0.76–1.22)	0.96 (0.76–1.23)	0.76
rs11556218					
T	504 (70.4)	621 (77.7)	1.00 (Ref)	1.00 (Ref)	
G	212 (29.6)	183 (22.8)	**1.43 (1.13–1.80)**	**1.44 (1.14–1.81)**	0.002
rs4072111					
C	560 (78.2)	616 (76.6)	1.00 (Ref)	1.00 (Ref)	
T	156 (21.8)	188 (23.4)	0.91 (0.72–1.16)	0.91 (0.72–1.16)	0.45

SNPs, single nucleotide polymorphisms; OR, odds ratio; CI, confidence interval.

^†^Adjusted by age and gender.
